# Silk Particle Production Based on Silk/PVA Phase Separation Using a Microfabricated Co-flow Device

**DOI:** 10.3390/molecules25040890

**Published:** 2020-02-17

**Authors:** Natalia Vargas Montoya, Rachel Peterson, Kimberly J. Ornell, Dirk R. Albrecht, Jeannine M. Coburn

**Affiliations:** Department of Biomedical Engineering, Worcester Polytechnic Institute, Worcester, MA 01609, USA; nvargasmontoya@wpi.edu (N.V.M.); rdpeterson@wpi.edu (R.P.); kjornell@wpi.edu (K.J.O.); dalbrecht@wpi.edu (D.R.A.)

**Keywords:** microfluidics, silk fibroin, particles, poly(vinyl alcohol), drug delivery, macrophages, phase separation

## Abstract

Polymeric particles are ideal drug delivery systems due to their cellular uptake-relevant size. Microparticles could be developed for direct injection of drug formulations into a diseased site, such as a tumor, allowing for drug retention and slow drug exposure over time through sustained release mechanisms. *Bombyx mori* silk fibroin has shown promise as a biocompatible biomaterial both in research and the clinic. Silk has been previously used to make particles using an emulsion-based method with poly(vinyl alcohol) (PVA). In this study, polydimethylsiloxane-based microfluidic devices were designed, fabricated, and characterized to produce silk particles through self-association of silk when exposed to PVA. Three main variables resulted in differences in particle size and size distribution, or polydispersity index (PDI). Utilizing a co-flow microfluidic device decreased the PDI of the silk particles as compared to an emulsion-based method (0.13 versus 0.65, respectively). With a flow-focusing microfluidics device, lowering the silk flow rate from 0.80 to 0.06 mL/h resulted in a decrease in the median particle size from 6.8 to 3.0 μm and the PDI from 0.12 to 0.05, respectively. Lastly, decreasing the silk concentration from 12% to 2% resulted in a decrease in the median particle size from 5.6 to 2.8 μm and the PDI from 0.81 to 0.25, respectively. Binding and release of doxorubicin, a cytotoxic drug commonly used for cancer treatment, with the fabricated silk particles was evaluated. Doxorubicin loading in the silk particles was approximately 41 µg/mg; sustained doxorubicin release occurred over 23 days. When the cytotoxicity of the released doxorubicin was tested on KELLY neuroblastoma cells, significant cell death was observed. To demonstrate the potential for internalization of the silk particles, both KELLY and THP-1-derived macrophages were exposed to fluorescently labelled silk particles for up to 24 h. With the macrophages, internalization of the silk particles was observed. Additionally, THP-1 derived macrophages exposure to silk particles increased TNF-α secretion. Overall, this microfluidics-based approach for fabricating silk particles utilizing PVA as a means to induce phase separation and silk self-assembly is a promising approach to control particle size and size distribution. These silk particles may be utilized for a variety of biomedical applications including drug delivery to multiple cell types within a tumor microenvironment.

## 1. Introduction

Novel polymeric particles are some of the most promising areas of drug delivery due to their cellular uptake-relevant size [1]. This drug delivery method is characterized as either a systemic or local delivery system [2]. Systemic delivery systems are primarily IV injections containing nanoparticles that circulate through blood vessels until they reach their treatment site. However, when designing a systemic delivery system, it is difficult to determine how much payload will reach the target as it might be cleared from the blood by the immune system, lungs, liver, or kidneys [3]. Unlike systemic delivery, local drug delivery is inserted or injected directly into or around the treatment site. Micron sized particles have been researched as a localized delivery system for many diseases as their size allow for higher drug loading and can remain in the treatment site preventing intravasation into blood vessels [4,5,6,7,8]. Hypervascularized liver tumors have been treated with locally delivered polymeric microparticles [e.g., sulfated poly(vinyl alcohol) particles loaded with doxorubicin (DEBDOX^®^) and sodium acrylate alcohol copolymer microspheres loaded with doxorubicin (Hepaspheres^®^)] [9,10,11].

The main requirement for a good polymeric delivery system is an appropriate polymer that will not harm the patient and can be engineered for drug loading and degradation [12]. *Bombyx mori* silk fibroin is a biocompatible biomaterial used in many biomedical applications due to its lack of toxicity and ability to be fabricated into several drug delivery form factors, including but not limited to particles [12,13,14,15,16,17,18,19]. By regulating the crystallinity of silk, the degradability can de designed for controlled release which is beneficial for drug delivery applications [20,21]. Phase separation, jet spraying, and lipid particle encapsulation have all been used to fabricate silk fibroin particles; however, the size of these particles is difficult to control and non-biologically relevant particle sizes need to be filtered out [19,22]. To obtain more controllable sizes and distributions, microfluidics has been applied to silk fibroin to produce particles [20,23,24].

Microfluidics is the behavior, control, and manipulation of sub-milliliter volumes of fluids [25]. At this scale, interfacial tension and capillary forces can be varied to change the behavior of fluids. Microfluidics has been used in silk particle formation by taking advantage of silk’s ability to β-sheet when exposed to organic solvents or high forces [20,23,24]. While these devices formed nano-sized [20,24] and high micron-sized particles [23], the particular devices were fragile and required technical expertise to reproduce. Once a device clogs or breaks, a new one would need to be made, resulting in the technical challenge of reproducing the placement of the capillaries or needles [26,27]. Another widely used method of microfluidic device manufacturing is soft lithography using polydimethylsiloxane (PDMS) [28].

In this work, we developed a silk particle fabrication technique that resulted in controlled particle size and particle size distribution using a manufactured PDMS microfluidic device. We investigated the impact of several process parameters on particle size and particle size distribution including flow rate, external phase concentration, internal phase concentration, and internal phase polymer molecular weight. The resulting silk particles were functionally evaluated for cytotoxicity, drug delivery, activity of the released drug, and macrophage uptake and activation.

## 2. Results and Discussion

### 2.1. Microfluidics Device Fabrication and Characterization

**Figure 1:**
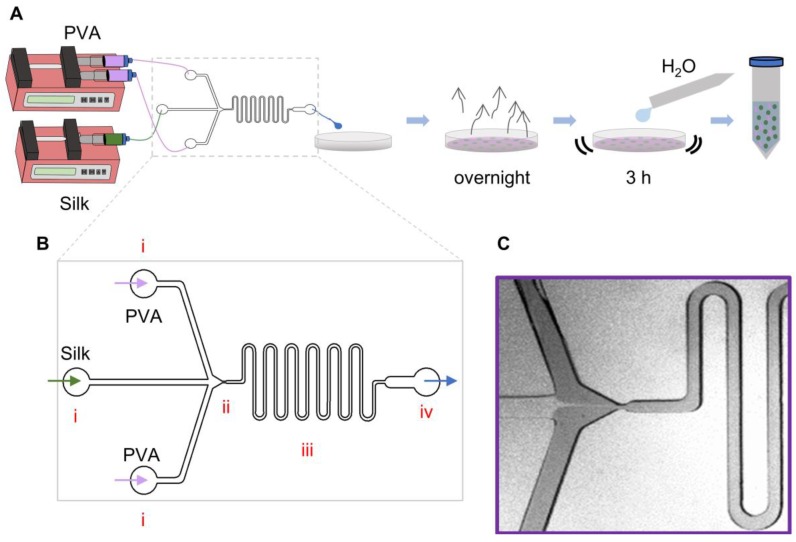
Schematic of the silk particle fabrication device. (**A**) Process for silk particle fabrication via co-flow and flow-focusing microfluidics device. The fluids (silk solution as the internal phase; PVA solution as the external phase) flow through the device using syringe pumps. The output is collected in a petri dish and allowed to evaporate overnight. The result film is dissolved to separate the silk particles and remove soluble polymers. (**B**) Larger schematic view of the flow-focusing device. The two solutions (the external phase: purple arrow; the internal phase: green arrow) flow into the device through the inlets (i) and meet at the flow-focusing channel device (ii). They then flow together through the serpentine (iii) until they exit the device through the outlet (iv; blue arrow). (**C**) Stereoscope image of the two solutions flowing through the device with the PVA solution dyed with food coloring to provide contrast. The flow-focusing channel device width, the internal phase flow rate, polymer concentrations, and silk molecular weight were the process variables for this study.

**Figure 2:**
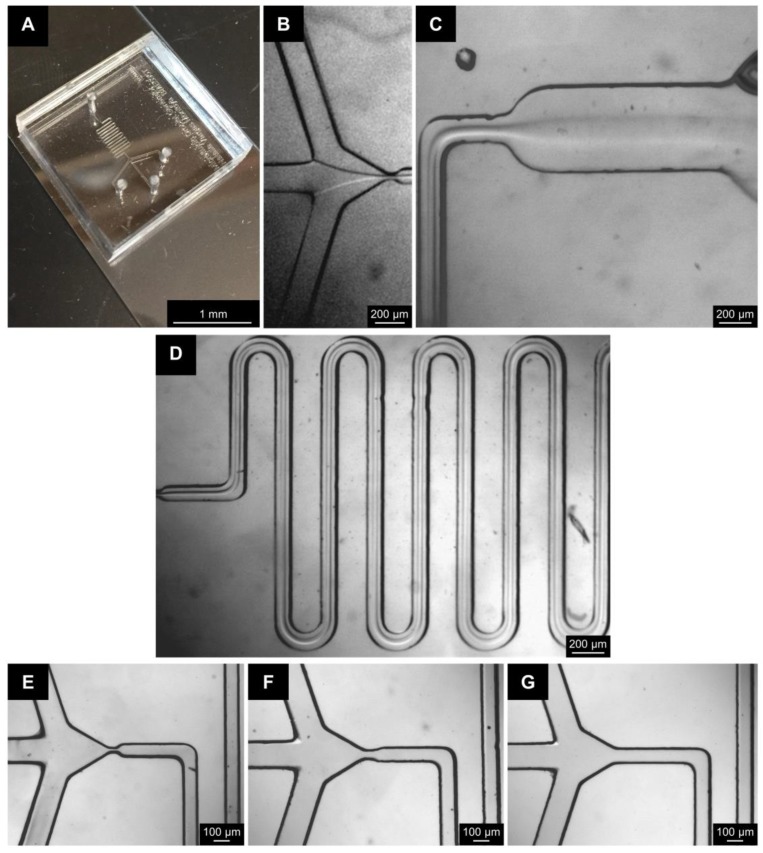
Images of the devices with and without fluid flow. Image of (**A**) a completely fabricated microfluidics device, (**B**) fluid flow at the point where the two fluids contact; (**C**) the outlet of the serpentine, and (**D**) fluid flowing through the serpentine. Three different configurations were utilized: two flow-focusing channels of (**E**) 20 µm and (**F**) 50 µm, and (**G**) a 100 µm co-flow channel. Note that dust is on the device, not within channels. Images taken with a stereo microscope.

To fabricate the fluidics system for this study (Figure 1A), a negative wafer was developed using photolithography techniques. The parameters used for fabricating the negative wafer were determined using information provided by the photoresist manufacturer. This wafer can be reused multiple times to make many devices with the same dimensions. The microfluidic devices were fabricated by curing PDMS onto the wafer. The individual PDMS molds were plasma bonded onto glass slides to generate the final microfluidic devices (Figure 2A). The internal channel dimensions were measured by making cross sectional splices of the devices (Figure S1); these measurements were compared to the theoretical dimensions in the design developed using DraftSight (Table 1).

To characterize the theoretical fluid flow properties within the device, the Reynolds (Re) and Dean (Dn) numbers for each distinct region (Figure 1B and Figure S1) of the device and each fluid were calculated. Transition from laminar flow to turbulent occurs when Re > 2300 in a straight tube and Dn > 40 in a curved tube. The calculations show that for all the fluids flowed through the device, the flow would exhibit a stable, unidirectional, and nonturbulent flow since all Re < 2300 and Dn < 40 (Table 2). This was further confirmed with images taken during device runs (Figure 1C and Figure 2B–D).

### 2.2. Particle Fabrication Using PVA/Silk: Comparing Emulsion Method and Co-Flow Method

**Figure 3:**
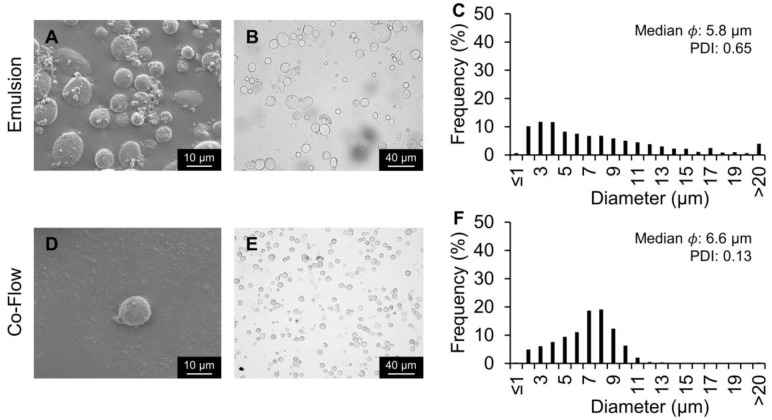
Silk particles fabricated via the emulsion method and a co-flow microfluidics device method. The same ratio of 5% PVA and 7% silk was used (3 and 2.4 mL, respectively). (**A**,**D**) SEM and (**B**,**E**) brightfield microscopy images of silk particles. (**C**,**F**) Silk particle size distribution, median, and PDI measured via image analysis.

Silk, poly(d,l-lactide-co-glycolide), and other polymeric particles have been produced using PVA-based approaches [23,29,30,31,32]. Silk particles can be produced via a batch emulsion process using PVA. When the PVA and silk interact, phase separation and self-association of the silk occurs forming intermolecular and intramolecular hydrogen bonding and a secondary structure with β-sheets. This β-sheeting allows for structural stabilization of the silk molecules effectively making them insoluble forming rigid silk particles with stable size and shape [18]. To determine whether the microfluidic device would be beneficial in the fabrication of silk particles, the silk particles developed by the co-flow microfluidic device were compared to a standard emulsion method [18]. The same ratio of PVA and silk (3 and 2.4 mL, respectively) was used in both the emulsion and co-flow methods. While initially expecting the device to facilitate droplet formation of the silk phase as previously reported [20], droplets were not observed within the co-flow device (Figure 2B–D). Instead, the device allowed for controlled silk/PVA interaction with more uniform and consistent exposure of the silk to the PVA compared to emulsion processing. SEM and brightfield images confirmed that both methods produced silk particles (Figure 3). Additionally, it demonstrated that, while the median particle size did not differ between the two silk particle fabrication methods (5.8 µm for emulsion and 6.6 µm for co-flow), the PDI was lower for the co-flow fabrication method (0.13) as compared to the emulsion silk particle fabrication method (0.65) suggesting a more homogeneous particle size distribution.

To determine the effect of flow-focusing geometry on particle size and PDI, silk particles were fabricated using 20 µm and 50 µm flow-focusing devices (Figure S3). No differences were observed between the co-flow fabrication method as compared to the two flow-focusing fabrication methods when maintaining the flow rate of the internal phase constant. Since there were no differences observed when changing the width of the flow-focusing channel device and literature has demonstrated a higher probability of fabricating smaller silk particles with a more narrow channel [33], a 20 µm flow-focusing device was used for the remainder of the experiments, unless stated otherwise.

### 2.3. Silk Particles Fabricated By Varying Flow Rates

**Figure 4:**
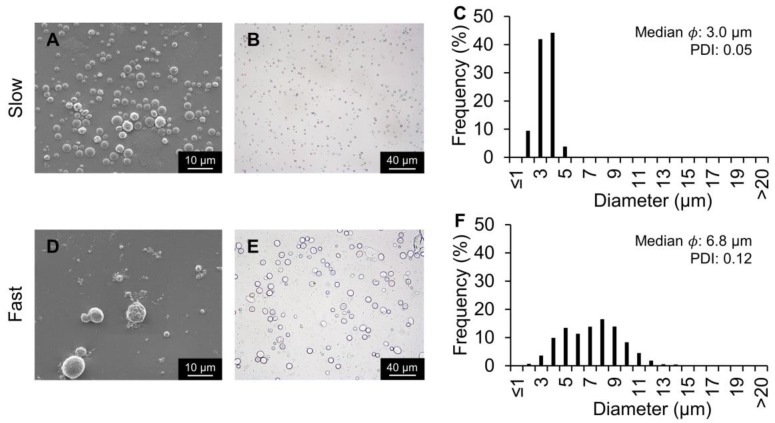
Silk particles fabricated using the 20 µm flow-focusing microfluidics device and varying the dispersed phase flow rate. The internal phase flow rate was either 0.06 mL/h (slow) or 0.8 mL/h (fast). The internal phase contained silk at 7%; the external phase contained PVA at 5%. (**A**,**D**) SEM and (**B**,**E**) brightfield microscopy images of silk particles. (**C**,**F**) Silk particle size distribution, median, and polydispersity index (PDI) measured via image analysis.

To determine how the internal phase flow rate affected silk particle fabrication, the flow rate of the internal phase was decreased from 0.8 to 0.06 mL/h (Figure 4). Flow rates below 0.06 mL/h resulted in backflow of the internal phase towards the inlet. The median size decreased with the lower flow rate (from 6.8 µm to 3.0 µm) as did the PDI (from 0.12 to 0.05). The 50 µm flow-focusing channel device and the co-flow device were evaluated with the slower internal phase flow rate to determine whether the reduced silk particle size and PDI was consistently observed (Figure S3 and S4). Decreasing flow rate for all devices resulted in decreased silk particle size and PDI. These findings are consistent with previous co-flow devices used for silk particle production [20]. This could be because as the silk flow rate is lowered, the ratio of PVA to silk increases resulting in a higher surface area instantly affected by the phase separation.

**Figure 5:**
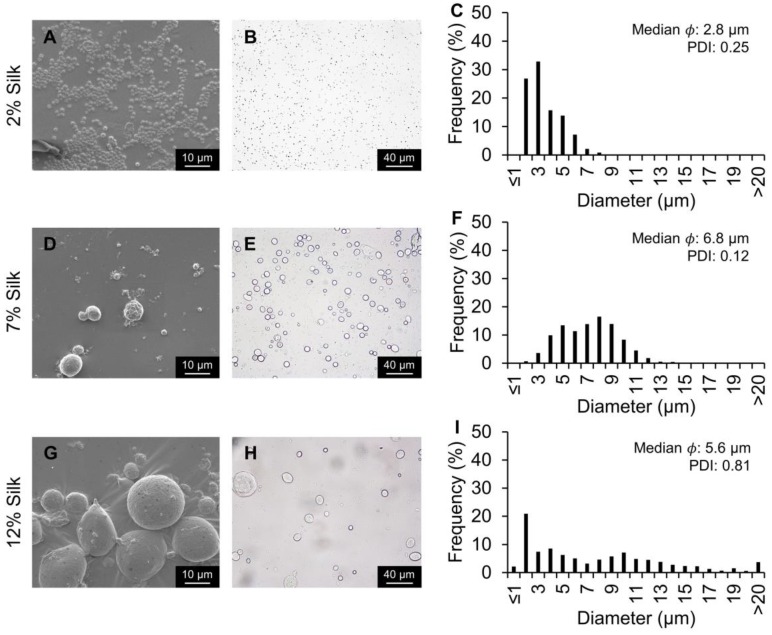
Silk particles fabricated using the 20 µm flow-focusing microfluidics device and varying the dispersed phase silk concentration. The dispersed phase concentration was either 2%, 7%, or 12% silk at 0.8 mL/h. The external phase concentration was 5% PVA. (**A**,**D**,**G**) SEM and (**B**,**E**,**H**) brightfield microscopy images of silk particles. (**C**,**F**,**I**) Silk particle size distribution, median, and PDI measured via image analysis.

To determine the effect of changing the internal phase concentration on silk particle fabrication, 2%, 7%, and 12% silk solutions were evaluated at the fast flow rate (Figure 5). The median silk particle size decreased from 5.6 to 2.8 µm for the 12% the 2% silk solutions, respectively. Additionally, the PDI decreased with decreasing silk concentration (0.81, 0.12, and 0.05 for 12%, 7%, and 2% silk solutions, respectively). To determine the effect of changing the external phase concentration on silk particle fabrication, 1%, 5%, and 10% PVA solutions were evaluated (Figure S5). When the PVA concentration was increased from 5% to 10%, a higher PDI (0.12 to 0.26) was observed. When the PVA concentration was decreased to 1% a predominantly non-spherical morphology was observed, and few silk particles were present making the quantification of the particle size inaccurate. This is consistent with other silk particle formation methods which showed that when PVA is not added, unpredictable silk aggregations were observed [34,35]. Silk films are formed by casting the aqueous silk solution [35]. Therefore, when there is insufficient PVA present to ensure phase separation, the silk will dry on the petri dish and result in film fragments when dissolved.

### 2.4. Silk Particles Fabricated By Varying Silk Molecular Weight

After determining the effect of silk and PVA concentration on silk particle size and PDI, the impact of silk molecular weight on silk particle fabrication was evaluated. Two silk extract times were utilized to obtain the different average molecular weights: 30 min extracted (mE) to obtain the high molecular weight formulation and 120 mE to obtain the low molecular weight formation. Silk molecular weight did not affect silk particle size (6.8 µm versus 7.1 µm for low molecular weight versus high molecule weight) (Figure S6). However, lower silk molecular weight resulted in a lower PDI (0.12 versus 0.21 for low molecular weight versus high molecule weight).

### 2.5. Doxorubicin Binding and Release from Silk Particles

**Figure 6:**
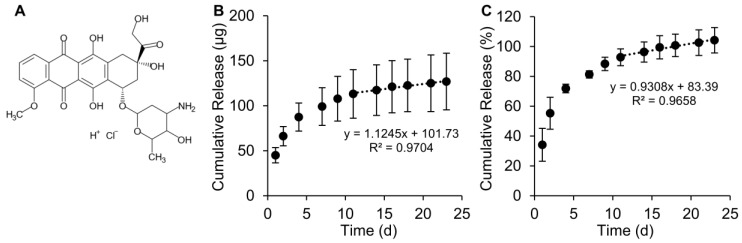
Release of doxorubicin from silk particles. (**A**) Structure of doxorubicin HCl used for the drug release experiments. Cumulative (**B**) mass and (**C**) percent release over 23 days with linear release of ~1.1 µg/day after day 11. Data are presented as the mean ± S.D. from three independent experiments each with three separate samples.

Next, we examined the use of the silk particles for drug release. Doxorubicin, below its pKa of ~9.93, is positively charged; silk, above its isoelectric point of ~4.2, is negatively charged. The mechanism of initial doxorubicin/silk interaction has been previously reported to be through electrostatic interaction [36]. Drug loading was performed via an adsorption from solution methods [16,17,36,37,38]. Doxorubicin, a cytotoxic chemotherapy agent, was loaded into silk particles fabricated using the 20 μm flow-focusing device and the fast flow rate, resulting in silk particles with a median size of 6.8 μm. This silk particle fabrication procedure was chosen because it resulted in a high silk particle yield based on the silk concentration and the fast flow rate. Additionally, the larger silk particle size allowed for rapid post-fabrication washing higher-throughput experimentation. The average doxorubicin loading was 41 ± 8.3 μg/mg of silk, with approximately 100% loading efficiency. The drug loading capacity observed here was similar to previously reported doxorubicin loading into silk nanoparticles [16]. Sustained doxorubicin release was observed through 23 days (Figure 6) with a linear release of 1.1 µg doxorubicin per d after day 11. The mechanism of doxorubicin release is likely through competitive binding of cations present within the PBS and the anionic groups in silk fibroin occupied by doxorubicin. Previously, doxorubicin binding was inhibited by the presence of sodium chloride in the adsorption solution [36]. Diffusion likely plays a role as well, though likely to a limited extent as silk materials loaded with doxorubicin and stored in water do not exhibit noticeable drug release over extended periods of time [36,37,38]. In the clinic, the doxorubicin dosing cycle is 28 d [39], which is similar in length to the 23 days release. Therefore, it is plausible that these doxorubicin-releasing silk particles could be explored as an inter-tumoral drug delivery system to control the growth of solid tumors. Within the tumor microenvironment, the pH is often lower than normal physiological conditions due to increased metabolism and build-up of acidic, metabolic byproducts. Previously literature has reported an increased in doxorubicin release from silk particles under acidic pH conditions [16,17,24]. Additionally, Chiu et al. reported increased doxorubicin release within mouse neuroblastoma tumors as compared to neutral pH conditions in vitro [38]. Therefore, increased doxorubicin release from the silk particles developed here would be expected within an acidic tumor microenvironment. Additionally, doxorubicin release may be increased due to the metabolic enzymes within the intracellular and extracellular space of the tumor microenvironment.

### 2.6. KELLY Neuroblastoma Cell Response to Silk Particles

**Figure 7:**
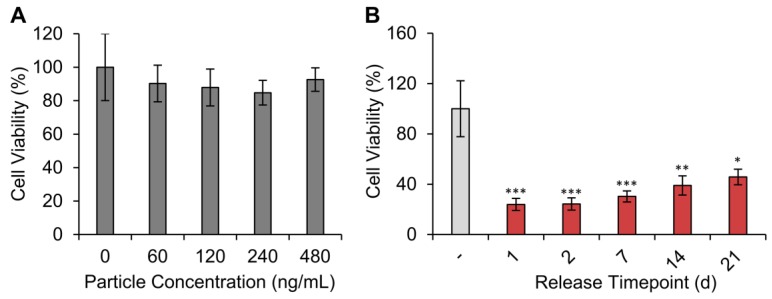
In vitro cytotoxicity of silk particles against the human neuroblastoma cells (KELLY cell line). (**A**) KELLY cells were exposed for 2 d to varying concentrations of silk particles (median size: 6.8 µm). No change in cell viability was observed. Data are presented as the mean ± S.D. from three separate samples. (**B**) KELLY cells were exposed for 2 d to doxorubicin release medium from day 0–1, 1–2, 5–7, 11–14, and 18-21 at a 10-fold dilution in cell culture medium. All time points resulted in statistically significant reduction in cell viability compared to the untreated control (-). Data are presented as the mean ± S.D. from three independent experiments each with three separate samples. Two-way ANOVA followed by Tukey post-hoc test; * *p* < 0.05, ** *p* < 0.01, *** *p* < 0.001 as compared to untreated control.

We next sought to determine whether the silk particles induced toxicity to mammalian cells. For these experiments, we used the same silk particle formulation as used for the drug release studies. KELLY cells were exposure to silk particle concentrations ranging from 60 to 480 ng/mL for 48 h. All silk particle concentrations evaluated did not induce significant changes in cell viability (Figure 7A).

To evaluate cytotoxicity induced by the doxorubicin released from the silk particles, release studies were performed aseptically in PBS. The sterile PBS with release doxorubicin on day 0–1, 1–2, 5–7, 11–14, and 18–21 of release were diluted 10-fold in cell culture medium (drug concentration provided in Table S1). The KELLY cells were exposed to the 10-fold diluted solutions for 2 d. All release time points induced significant cytotoxicity when compared to non-drug treated cells (Figure 7B). The initial doxorubicin release from day 1 resulted in the greatest cytotoxicity, as expected based on the drug release profiles. A slight increase in cell viability with increasing release time was observed, though not statistically different, suggesting continuous cytotoxicity over the entire experimental range.

### 2.7. Association of Silk Particles with KELLY Neuroblastoma Cell

Next, we sought to evaluate silk particle association or internalization by KELLY cells. Since KELLY cells are non-phagocytotic cells, the smallest silk particles fabricated (2% silk, 5% PVA, 20 µm flow-focusing channel device, and fast flow rate) with a median size of 2.8 μm (min: 1.0 µm; max: 7.1 µm) were used. FITC-conjugated silk particles were incubated with KELLY cells for 2, 4, 8, and 24 h (Figure S7). While particle uptake or attachment was observed at 24 h, it was difficult to determine the extent of uptake via routine fluorescent microscopy. To better visualize the uptake, confocal images were taken of cells exposed to silk particles after 24 h. The 3D projection images (Figure S8) and Z-stacks and 3D rendering videos (Video S1) images better show the inside of the cells. Though few silk particles were associated with the cells, we measured the silk particle size to be 4.4 ± 1.2 µm.

### 2.8. Interaction of Silk Particles with Human THP-1-Derived Macrophage

**Figure 8:**
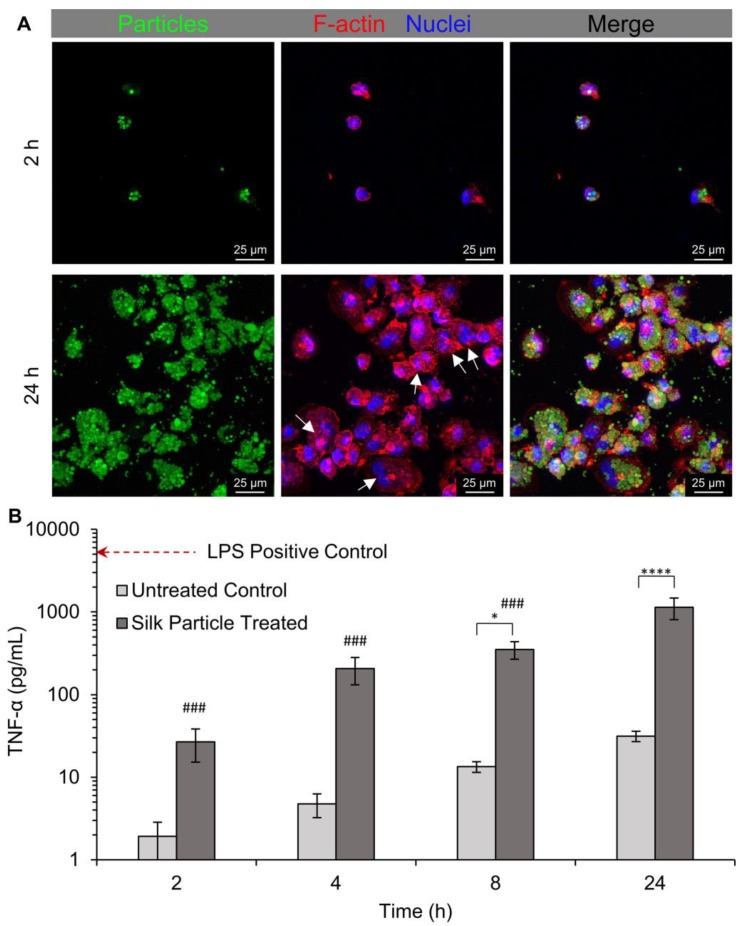
The 3D projection and tumor necrosis factor-α (TNF-α) secretion of the macrophage exposed to silk particles (median size: 2.8 µm). Cells were exposed to silk particles for 2, 4, 8, and 24 h. (**A**) Confocal images were taken after 2 and 24 h of exposure. White arrows indicate macrophages that appeared to be multi-nucleated indication macrophage fusion. (**B**) The TNF-α secreted in the media was quantified at all time points and lipopolysaccharide (LPS) was used as a positive control, represented by the horizontal line. All samples are statistically different than the LPS control (p < 0.0001). Asterisks indicate statistical significance between silk particle treatment and the untreated controls at the respective time points (* *p* < 0.05, **** *p* < 0.0001). Pound indicates statistical significance compared to the 24 h time point when comparing silk particle groups (### *p* < 0.0001).

Silk particle uptake by human THP-1-derived macrophages was qualitatively evaluated via fluorescent and confocal microscopy imaging. The same silk particles used for the KELLY cell particle association experiments were used for the THP-1-derived macrophage experiments. Differentiation of THP-1 monocytes to macrophage was confirmed via the transition from a suspension cell phenotype to an attached cell phenotype (Figure S9) and upregulation of the macrophage marker CD68 (Table S2) [40,41]. Once differentiation was confirmed, silk particle interaction with the macrophages was examined. The macrophages were exposed to FITC-conjugated silk particles for 2, 4, 8, and 24 h. Interaction, either uptake or association, was observed at all timepoints evaluated with a qualitative increase correlating with exposure time (Figure S10). To better visualize the internalization of the silk particles, confocal images were taken of cells exposed to silk particles for 2 and 24 h (Figure 8A and Video S2). After 24 h, the cells appeared to be multi-nucleated indicating macrophage fusion (Figure 8A, white arrows). This shows that while there is some residual PVA, less than 5 wt%, [18] its effect on surface hydrophobicity [29,30] was not enough to prevent uptake by macrophages. The average silk particle size that appears to be internalized into the macrophages was 3.1 ± 0.6 µm. Previous publications have shown that this particle size is within the ideal size range for phagocytosis [42,43].

Next, we sought to quantitatively assess macrophage activation by silk particle exposure and uptake (i.e., phagocytosis-induced macrophage activation). TNF-α secretion, a classical marker of macrophage activation, was measured at each time point (Figure 8B). The TNF-α secreted from the 2 h and 4 h untreated control was negligible. However, the TNF-α secretion of silk particle treated cells was statistically higher than the untreated controls after 8 and 24 h of exposure with 24 h exhibited the highest TNF-α secretion. The positive control, THP-1-derived macrophages treated with LPS, exhibited the highest TNF-α secretion. This test verifies classical macrophage activation in response to silk particle exposure. The confocal microscopy indicates that the silk particles were internalized by the macrophages likely through phagocytosis. Phagocytosis results in macrophage activation and TNF-α secretion as an initiation of the inflammatory response.

## 3. Materials and Methods

### 3.1. Silk Fibroin Solution Preparation

Silk fibroin was extracted from *Bombyx mori* silkworm cocoons as previously described [13]. Briefly, silk cocoons were cut into 1 cm × 1 cm pieces and boiled in 0.02 M sodium carbonate (Sigma, St. Louis, MO, USA) for 30 min or 120 min to remove the sericin protein. Additionally, this extraction process hydrolyzes the silk fibroin into shorter silk fragments. The resulting silk fibroin fibers were dried overnight and dissolved in 9.3 M lithium bromide (Sigma, St. Louis, MO, USA) for 3 h at 60 °C followed by dialysis against ultrapure water (3.4 kDa MWCO Fisher Scientific, Hampton, NH, USA) for 2.5 d with at least 7 water changes. The resulting aqueous silk fibroin solution (referred to as “silk” from here on) was stored at 4 °C. The average molecular weight for 30 min extracted (30 mE) and 120 min extracted (120 mE) silk has previously been determined to be approximately 210 kDa and 67 kDa, respectively [44].

### 3.2. Poly(vinyl alcohol) Solution Preparation

Poly(vinyl alcohol) (PVA) is an U.S. Food and Drug Administration approved polymer that has widely been used in drug formation [18]. A 10% (*w*/*v*) solution of PVA (99% hydrolyzed, SigmaAldrich, St. Louis, MO) was generated by dissolving PVA in ultrapure water overnight at 60 °C with continuous stirring. The solution was stored at room temperature, and if needed, diluted to the desired concentration.

### 3.3. Fabrication of Microfluidics Device

Using DraftSight (v. 2017.1, Dassault Systèmes, Vélizy-Villacoublay, France), co-flow and flow-focusing devices with three inlets (1 mm wide circle and 200 µm wide channels), a 100 µm wide serpentine channel, and one outlet were designed. The two outer inlets were used for the external phase, the PVA solution, while the center inlet was used for the internal phase, the silk solution. Photolithography and soft lithography techniques were used to make the reproducible devices [45]. First, a master silicon wafer was fabricated using photolithography. The wafer was coated with a 100 µm thick high contrast negative photoresist layer (SU8 2035) (MicroChem, Westborough, MA, USA), covered with a photomask of desired features, and cured using UV rays. After fabricating the master wafer, soft lithography was used to make the individual PDMS devices. PDMS (Sylgard^TM^ 184, Dow Corning, Midland, MI, USA) was cured on the wafer and the individual devices cut out. These PDMS devices were plasma bonded onto glass slides and incubated at 60 °C overnight to restore the hydrophobicity of the PDMS.

### 3.4. Microfluidics Device Characterization

To determine the dimensions of the device, the devices were cut between the inlets and the flow-focusing channel device, imaged, and measurements made of the channel height and width (Figure S1). Additionally, the widths of the flow-focusing regions and diameter of the inlets were measured. To characterize the theoretical flow properties, the *Re* and *Dn* numbers were calculated using the following equations:

(1) $$Re=\frac{\rho VD_{H}}{\mu }$$

(2) $$D_{H}=\frac{4A}{P}$$

(3) $$Dn=Re\sqrt{\frac{D_{H}}{2R_{c}}}$$

where ρ is density of the solution, *V* is velocity of the solution, *D_H_* is the hydraulic radius, μ is dynamic viscosity, *A* is the area, *P* is the perimeter, and $R_{c}$ is the radius of serpentine curvatures. The density was determined by weighing 1 mL of each solution with a positive displacement pipette. The velocity was calculated from the flow rate of the syringe pump and the measured area. The dynamic viscosity of each solution was measured using a MCR302 WESP Rheometer (Anton Paar, Ashland, VA) with a 50 mm parallel plate for 30 mE silk and 50 mm cone with a 0.5° angle for the other solutions. The theoretical dimensions were calculated from the original design and the actual height was measured from microscopy images. For calculating the dimensionless numbers at the flow-focusing channel device, the *Re* and *Dn* were calculated as if the solutions filled the whole channel (using the actual channel measurements), if both solutions had laminar flow, *Re* < 2,300 and *Dn* < 40, it was assumed that the three streams (two PVA and one silk) flowing together exhibited laminar flow which was confirmed via visual inspection of the flow fields.

### 3.5. Silk Particle Fabrication Via Microfluidics Device

All solutions were filtered through 40 µm strainers before use to remove any debris that might clog the device. Different variables were tested to determine how they affected the resulting silk particles. Device designs with different channel widths were examined, a standard 100 µm co-flow (Figure 2G) and two flow-focusing devices (20 µm and 50 µm, Figure 2E,F). Flow rates evaluated were 0.8 mL/h (fast) and 0.06 mL/h (slow) for the internal phase, silk solution. The external phase, PVA solution, was maintained at 1 mL/h total for all silk particle fabrication runs. Solution concentration evaluated were 2%, 7%, and 12% (*w*/*v*) for the internal phase and 1%, 5%, 10% (*w*/*v*) for the external phase. Flow rates of the external and internal phases could not be decreased further (while maintaining the other constant) without the observation of backflow into the opposite phase. Flow rates could not be increased further without the device failing between the PDMS and the glass slide (limitation of the plasma bonding). Two different silk extraction times were evaluated (30 mE and 120 mE). Experiments used 120 mE silk except when silk molecular weight was the variable under investigation. The output of the microfluidics device flowed into a 60 mm petri dish for 3 h and the collected solution was allowed to air dry for 24 h. The dried material was suspended in ultrapure water via gentle shaking for 3 h resulting in dissolution of the PVA with silk particulates in suspension. The silk particle suspension was centrifuged at 18,000× *g* for 30 min at 4 °C followed by removal of the supernatant. The pellet was resuspended in ultrapure water and sonicated (Branson Sonifier 450, North Billerica, MA) at 20% output for 30 S. Centrifugation and resuspension steps were repeated three times and the silk particles stored at 4 °C.

### 3.6. Particular Fabrication Via Batch Emulsion Method

To compare to a standard silk/PVA particle fabrication method, particles were fabricated using a silk/PVA emulsion method (Figure S2) [18]. The same total volume of 7% silk and 5% PVA as would flow through the microfluidic device at the fast rate were mixed (2.4 mL silk and 3 mL PVA), poured into a petri dish, and allowed to air dry for 24 h. The dried material was processed to obtain the final silk particles and stored as outlined in the Silk particle fabrication via microfluidics device section.

### 3.7. Silk Particle Characterization

The silk particles were imaged using a scanning electron microscopy (SEM, JEOL 7000D, JEOL, Peabody, MA) and brightfield microcopy (Leica DMLB2, Leica, Buffalo Grove, IL). For particle sizing, a total of 30 images were taken for each experiment, three independent experiments with five brightfield images taken at 40x, for the representative image and sizing, and 100x, for sizing smaller particles. The silk particle diameters were determined using ImageJ. Since the particle size distribution of each formulation was right skewed, not exhibiting a normal distribution, the median particle size was calculated and reported here. To calculate the dispersity of the particle size in each sample, the polydispersity index (*PDI*) [46] of each sample was calculated, as follows:

(4) $$PDI={\left(\frac{standard\;deviation}{median}\right)}^{2}$$

*PDI* is a measure of the size range in the samples and increases with *standard deviation*. *PDI* < 0.1, indicates a sample range that is monodisperse, 0.1 < *PDI* < 0.4, moderate polydisperse, and *PDI* <0.4 broadly polydisperse. To prepare the samples for SEM imaging, the silk particles were dried on copper tape on a SEM pedestal and sputter coated with gold (25 mA, 60 s).

### 3.8. Doxorubicin HCl Loading and Release

To test the drug loading and release potential of the silk particles, 3 mg of particles fabricated using the 20 µm flow-focusing channel device, 7% 120 mE silk at 0.8 mL/h, and 5% PVA at 1 mL/h were incubated in 1 mL of 120 $\frac{µg}{mL}$ doxorubicin HCl (LC Laboratories, Woburn, MA), a common chemotherapy drug, in water for three days [16,37,38]. We will refer to doxorubicin HCl as doxorubicin from here on. The silk particles were centrifuged and the visible light absorbance at 485 nm was measured on a SpectraMax 190 microplate reader (Molecular Devices, San Jose, CA). A standard curve of known doxorubicin concentrations and the following equation was used to determine the doxorubicin loading:

(5) $$Drug\;bound\;\left(\frac{µg}{mg}\right)=\frac{\left[initial\;drug\;concentration\;-final\;drug\;concentration\right]\;\left(\mu g\right)}{amount\;of\;silk\;\left(mg\right)}$$

Loading efficiency was determined using the following equation:

(6) $$Loading\;efficiency\;\left(\%\right)=\frac{Drug\;bound\;\left(\frac{µg}{mg}\right)*3\;mg}{120\;\frac{µg}{mL}*1\;mL}\times 100\%\;Eqn\;5$$

To determine doxorubicin release, the drug-loaded silk particles were suspended in phosphate buffered saline (PBS, pH 7.4) and incubated at 37 °C. At periodic time points, the sample was centrifuged for 15 min at 16,000× *g*, and the PBS removed and replaced with fresh PBS. The absorbance of the release supernatant was measured to determine the doxorubicin release.

### 3.9. Fluorescent Labeling of Silk Particles

Silk particles fabricated using the 20 µm flow-focusing channel device, 7% 120 mE silk at 0.06 mL/h, and 5% PVA at 1 mL/h were conjugated with FITC (Acros Organics, Geel, Belgium). Silk particles (240 ng/mL) were incubated in 0.1 M Na_2_CO_3_-NaHCO_3_, pH 9, with 0.5 µL of 1 mg/mL FITC per mL. After 3 h, the silk particles were washed with ultrapure water and sterilized using 70% ethanol.

### 3.10. Cell Culturing

KELLY neuroblastoma cells (Sigma, St. Louis, MO, USA) and THP-1 monocytes (Sigma, St. Louis, MO, USA) were maintained at 5% CO_2_ and 37 °C Cell culture medium for both cell types was Roswell Park Memorial Institute 1640 (RPMI) media supplemented with 10% (v/v) fetal bovine serum (FBS), 100 IU/mL penicillin, 100 μg/mL streptomycin, and 2 mM L-glutamine. KELLY cells were trypsin passaged once 80% confluence was reached. THP-1 cells were grown as suspension culture maintained between 200,000 and 800,000 cells/mL to maintain their paracrine signaling.

### 3.11. Direct Silk Particle Exposure of KELLY Cell

Silk particles used to evaluate changes in cell viability upon silk particle exposure were fabricated using the 20 µm flow-focusing channel device, 7% 120 mE silk at 0.8 mL/h, and 5% PVA at 1 mL/h (same as for the doxorubicin loading and release experiments). To observe the effect of the released doxorubicin, 10,000 cells/per well were seeded in 96-well plates and allowed to recover for one day. To confirm that the unloaded silk particles were not toxic to the cells, the cells were incubated with silk particles at 60, 120, 240, and 480 ng/mL in medium for 2 d. Cell viability to silk particles was evaluated via a resazurin conversion assay.

### 3.12. Cytotoxicity of Release Doxorubicin

To determine the cytotoxicity of the drug released by the silk particles, KELLY cells were incubated for 48 h in cell culture medium containing 10-fold diluted drug release medium from days 0–1, 1–2, 4–7, 11–14, and 18–21. Cell viability for drug release supernatant treatment was evaluated via a resazurin conversion assay.

### 3.13. KELLY Cell Uptake of Silk Particles

Silk particles used for all cell association, surface or uptake, experiments were fabricated using the 20 μm flow-focusing device and the fast internal phase flow rate. KELLY cells were seeded onto 12 mm circular glass cover slips in 12-well plates at 100,000 cells/well and allowed to recover for 24 h. The medium was replaced with cell culture medium containing 250 ng/mL FITC-conjugated silk particles. After 2, 4, 8, and 24 h, the cover slips were removed, formaldehyde-fixed, and stained with Phalloidin 594-I (Abnova, Taipei, Taiwan) and Hoechst (Life Technologies, Carlsbad, CA, USA). Images were taken using an inverted fluorescent microscope (Eclipse E600, Nikon, Tokyo, Japan) and a spectral confocal microscope (TCS SP5, Leica, Buffalo Grove, IL). Z-stack images were taken with a 1 µm step size.

### 3.14. THP-1 Derived Macrophage Uptake of Silk Particles

To evaluate silk particle uptake by macrophages, human THP-1 monocyte cell line was used. THP-1 monocytes were differentiated to M0 macrophages as follows: 100,000 THP-1 cells were seeded on 12 mm circular cover slips and treated with 100 ng/mL PMA (Sigma, St. Louis, MO, USA). After 18 h of differentiation, the medium was replaced with 250 ng/mL FITC-labeled silk particles in fresh cell culture medium. The cells were formaldehyde-fixed and stained after 2, 4, 8, and 24 h. Imaging was performed using both fluorescent and confocal microscopy.

### 3.15. Tumor Necrosis Factor-α (TNF-α) Secretion

Medium from the macrophage silk particle uptake experiments were evaluated for TNF-α secretion via a TNF-α ELISA (R&D Systems, Minneapolis, MN, USA) following the manufacturer’s protocol. As a positive control, the THP-1-derived macrophages were exposed to 100 ng/mL lipopolysaccharides from *E. coli* (LPS; Sigma, St. Louis, MO, USA) [47].

### 3.16. Statistical Analysis

For non-drug loaded silk particle exposure to cell experiments, statistical significance was determined by a one-way analysis of variance (ANOVA) followed by Tukey honestly significant difference test. For cytotoxicity experiments, statistical significance was determined by a two-way ANOVA followed by Tukey honestly significant differences test in GraphPad Prism (Version 7.0; San Diego, CA). For TNF-α secretion experiments, statistical significance was determined by a one-way ANOVA followed by Dunnett’s multiple comparison. For all experiments, statistical differences were considered to be at *p* < 0.05.

## 4. Conclusions

The microfluidic devices fabricated using photolithography and PDMS soft lithography were beneficial for fabricating silk particles of different sizes by altering material and device characteristics. We demonstrated that using a microfluidic device decreases the size distribution, or PDI, of the silk particles when compared to a PVA/silk batch emulsion method. Decreasing the flow rate of the internal phase containing the silk solution and decreasing the silk solution concentration resulted in decreased particle size and PDI. The most dramatic differences in particle size and PDI was achieved by varying the silk from 2% to 12% with the median size and PDI increasing from 2.8 to 5.6 µm and 0.25 to 0.81, respectively. The smallest median particle size with the lowest PDI, or most monodisperse size distribution, was achieved with an internal phase flow rate of 0.06 mL/h containing 7%, 120 mE silk solution and an external phase flow rate of 1 mL/h containing 5% PVA (median size: 2.8 µm; PDI: 0.05). Additionally, we determined that the width of the flow-focusing channel device, PVA concentration, and silk molecular weight minimally impacted silk particle size and PDI. The silk particles were found to be non-toxic to neuroblastoma cells. The silk particles exhibited doxorubicin binding and release capabilities, which resulted in cytotoxicity against the neuroblastoma cells. To evaluate silk particle association and interaction with tumor microenvironment relevant cell types, the smallest silk particles fabricated were incubated with KELLY neuroblastoma cells and THP-1-derived macrophages. The silk particle associated with both cell types throughout the experimental time course. At 24 h, the THP-1-derived macrophages appeared to begin to fuse and contained internalized silk particles with a concomitant increase in TNF-α secretion. The macrophage response demonstrated a phagocytosis-induced sterile inflammatory response. These findings support this particle fabrication approach as a method to generate tunable silk particles with potential to effect multiple cell types within a tumor microenvironment through delivery of cytotoxic drugs or non-specific macrophage targeting.

## Figures and Tables

**Table 1:** Theoretical and measured dimensions of microfluidic device.

	Theoretical Dimension (µm)	Actual Dimension (µm)
Inlet Diameter	1000	1155 ± 127
Device Height	100	153 ± 23
Channel Width	200	231 ± 25
Serpentine Width	100	119 ± 8
100 µm co-flow point	100	116 ± 13
50 µm flow-focusing point	50	58 ± 6
20 µm flow-focusing point	20	23 ± 3

Data are presented as the mean ± standard deviation.

**Table 2:** Re and Dn numbers for the channels inside microfluidic device.

		Re Numbers					Dn Numbers
Sample	Viscosity (mPa*s)	Inlets	100 µm	50 µm FF	20 µm FF	Serpentine Linear Region	Serpentine Curved Region
2% Silk F ^a^	1.56	0.75			6.26	1.21	0.70
7% Silk F ^a^	3.37	0.33	0.55	1.11	2.79	0.54	0.31
12% Silk F ^a^	10.09	0.10			0.83	0.16	0.09
7% Silk S ^b^	3.37	0.03	0.04	0.08	0.21	0.04	0.02
6.3% Silk F ^a,c^	7.92	0.15			1.24	0.24	0.14
1% PVA	1.30	0.56			9.36	1.81	1.04
5% PVA	4.57	0.16	0.52	1.06	2.67	0.52	0.29
10% PVA	16.83	0.04			0.69	0.13	0.07

^a^ F denotes fast flow rate (0.8 mL/h); ^b^ S denotes slow flow rate (0.06 mL/h); ^c^ 30 min extract silk; all others are 120 min extracted silk.

## Supplementary Materials

The following are available online. Figure S1. Schematic of how the heights and widths were measured and regions where the Re and Dn numbers were calculated; Figure S2. Emulsion particle formation. PVA (5%) and silk (7%) were mixed together and poured onto a petri dish; Figure S3. Silk particles fabricated using the three different microfluidic device designs (co-flow 100 µm channel or flow-focusing 20 µm or 50 µm channel); Figure S4. Silk particles fabricated using the three different microfluidic device designs (co-flow 100 µm channel or flow-focusing 20 µm or 50 µm channel); Figure S5. Silk particles fabricated using the 20 µm flow-focusing microfluidics device and varying the external phase PVA concentration; Figure S6. Silk particles fabricated using the 20 µm flow-focusing microfluidics device and varying the silk molecular weight via varying the extraction time; Figure S7. Fluorescent microscopy images of KELLY cells exposed to silk particles through 24 h; Figure S8. Confocal microscopy 3D projection images of KELLY cell exposed to silk particles for 24 h; Figure S9. Brightfield images of THP-1 monocyte cell line and THP-1-derived macrophages; Figure S10. Fluorescent microscopy images of THP-1-derived macrophages exposure to silk particles through 24 h. Video S1. Video of confocal z-stack images of the KELLY cells exposed to silk particles for 24 h; Video S2. Video of the 3D rendered confocal images of the KELLY cells exposed to silk particles for 24 h; Video S3. Video of confocal z-stack images of the THP-1-derived macrophages exposed to silk particles for 2 h; Video S4. Video of the 3D rendered confocal images of the THP-1-derived macrophages exposed to silk particles for 2 h; Video S5. Video of confocal z-stack images of the THP-1-derived macrophages exposed to silk particles for 24 h; Video S6. Video of the 3D rendered confocal images of the THP-1-derived macrophages exposed to silk particles for 24 h.
