# Supplementary material for: Silk Particle Production Based on Silk/PVA Phase Separation Using a Microfabricated Co-flow Device
Source: Molecules. 2020 Feb 17;25(4):890. doi: 10.3390/molecules25040890 (PMC7070425; doi:10.3390/molecules25040890)
Supplement: Supplementary file 1 [file molecules-25-00890-s001.zip › Supplementary Materials/Supplementary Materials.docx]

**SUPPLEMENTARY MATERIALS**

**Silk particle production based on silk/PVA phase separation using a microfabricated co-flow device**

Natalia Vargas Montoya, Rachel Peterson, Kimberly J. Ornell, Dirk R. Albrecht, Jeannine M. Coburn^*^

Department of Biomedical Engineering, Worcester Polytechnic Institute, Worcester, MA, USA

**Total pages: 11**

**List of supplementary figures: 10**

Figure S1. Schematic of how the heights and widths were measured and regions where the Re and Dn numbers were calculated.

Figure S2. Emulsion particle formation. PVA (5%) and silk (7%) were mixed together and poured onto a petri dish.

Figure S3. Silk particles fabricated using the three different microfluid device designs (co-flow 100 µm channel or flow focusing 20 µm or 50 µm channel).

Figure S4. Silk particles fabricated using the three different microfluid device designs (co-flow 100 µm channel or flow focusing 20 µm or 50 µm channel).

Figure S5. Silk particles fabricated using the 20 µm flow-focusing microfluidics device and varying the external phase PVA concentration.

Figure S6. Silk particles fabricated using the 20 µm flow-focusing microfluidics device and varying the silk molecular weight via varying the extraction time.

Figure S7. Fluorescent microscopy images of KELLY cells exposed to silk particles through 24 h.

Figure S8. Confocal microscopy 3D projection images of KELLY cell exposed to silk particles for 24 h.

Figure S9. Brightfield images of THP-1 monocyte cell line and THP-1 derived macrophages.

Figure S10. Fluorescent microscopy images of THP-1 derived macrophages exposure to silk particles through 24 h.

**List of supplementary videos: 6**

**Video S1.** Video of confocal z-stack images of the KELLY cells exposed to silk particles for 24 h.

**Video S2.** Video of the 3D rendered confocal images of the KELLY cells exposed to silk particles for 24 h.

**Video S3.** Video of confocal z-stack images of the THP-1 derived macrophages exposed to silk particles for 2 h.

**Video S4.** Video of the 3D rendered confocal images of the THP-1 derived macrophages exposed to silk particles for 2 h.

**Video S5.** Video of confocal z-stack images of the THP-1 derived macrophages exposed to silk particles for 24 h.

**Video S6.** Video of the 3D rendered confocal images of the THP-1 derived macrophages exposed to silk particles for 24 h.


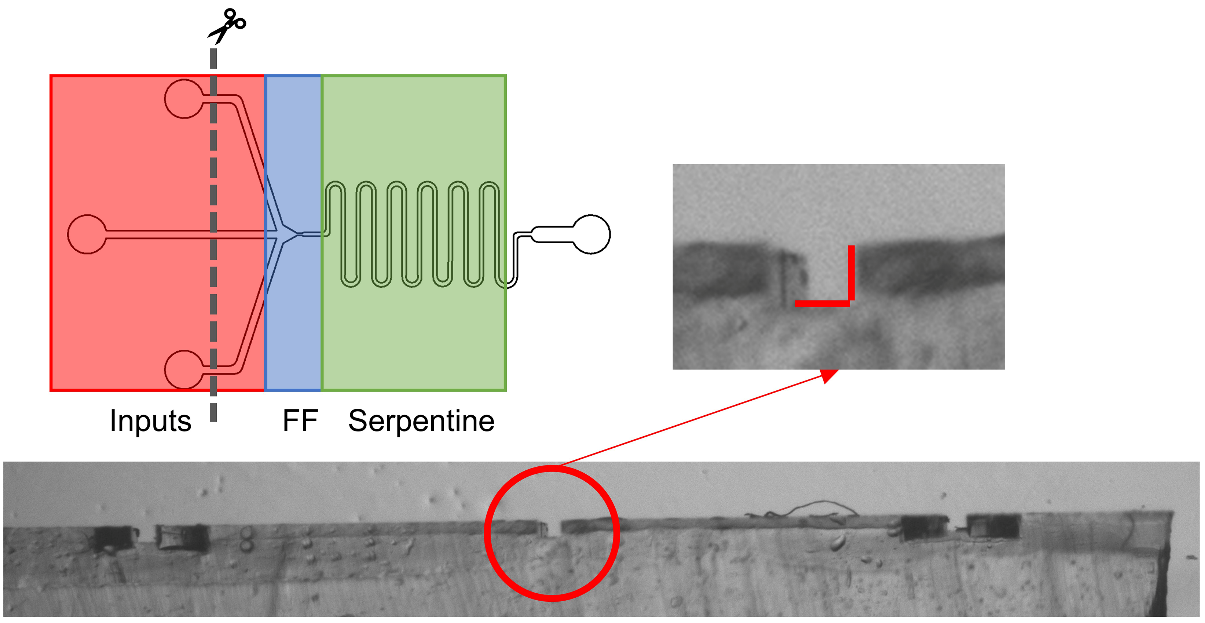


**Figure S1.** Schematic of how the heights and widths were measured and regions where the Re and Dn numbers were calculated. To measure the actual heights and widths of devices, one device from each wafer batch was sliced where the three channels are parallel (dotted line). The Re and Dn numbers were calculated at the distinct sections of the device; input (red), flow focusing channel (blue), and the serpentine channel (green). Images taken with a stereo microscope.


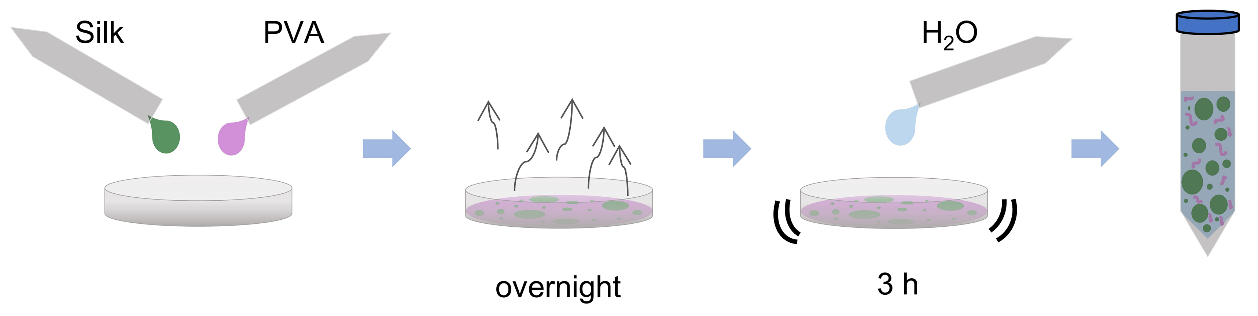


**Figure S2.** Emulsion particle formation. PVA (5%) and silk (7%) were mixed together and poured onto a petri dish. The water was evaporated overnight. To collect the particles, the resulting dry film was dissolved in water to remove soluble polymers (PVA and any potential non-particle associated silk).


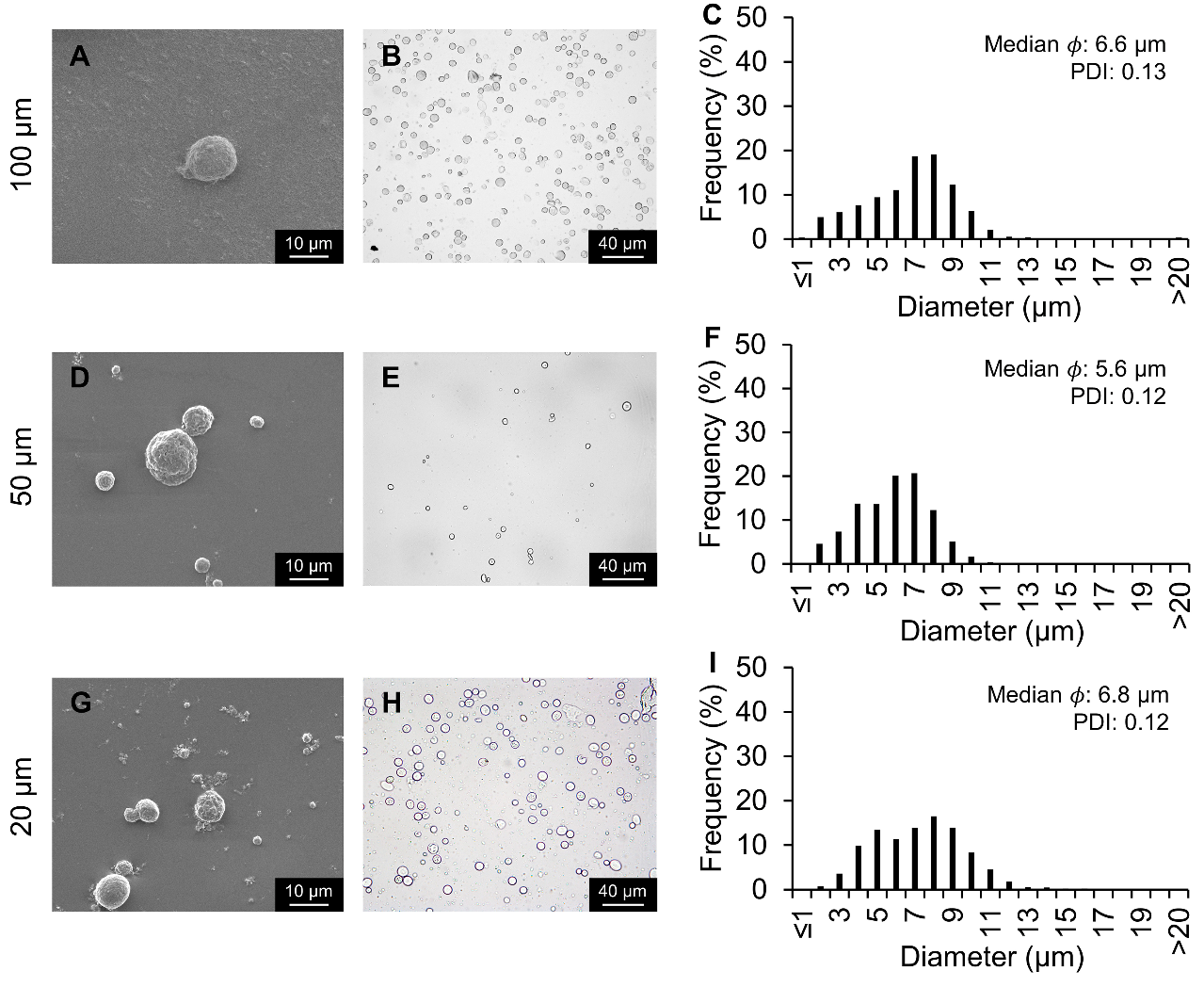


**Figure S3.** Silk particles fabricated using the three different microfluidic device designs (co-flow 100 µm channel or flow focusing 20 µm or 50 µm channel). The internal phase flow rate was 0.8 mL/h. The internal phase concentration was 7% silk; the external phase concentration was 5% PVA. (A,D,G) SEM and (B,E,H) brightfield microscopy images of silk particles. (C,F,I) Silk particle size distribution, median, and PDI measured via image analysis.


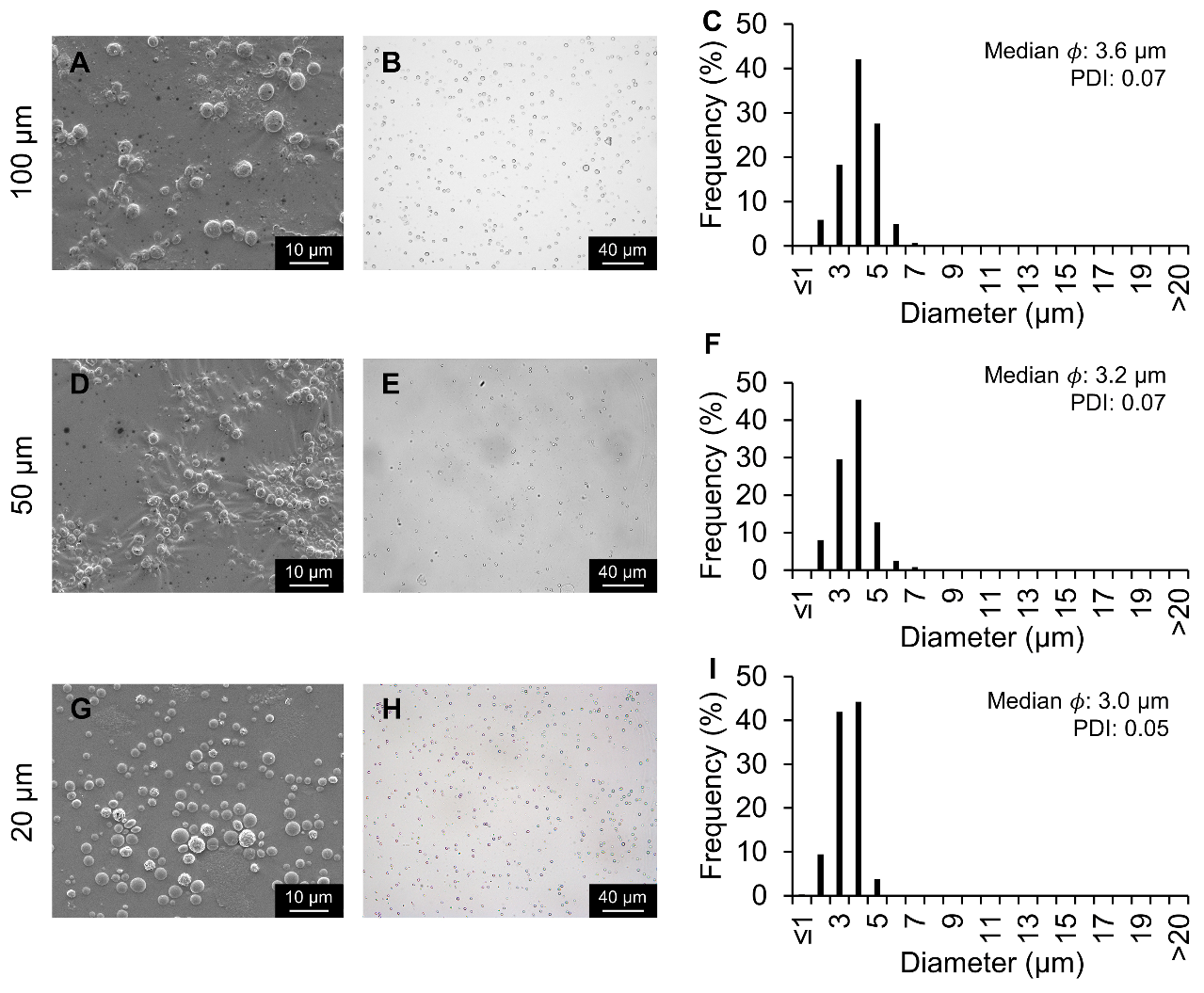


**Figure S4.** Silk particles fabricated using the three different microfluid device designs (co-flow 100 µm channel or flow focusing 20 µm or 50 µm channel). The internal phase flow rate was 0.06 mL/h (7% silk). The external phase concentration was 5% PVA. (A,D,G) SEM and (B,E,H) brightfield microscopy images of silk particles. (C,F,I) Silk particle size distribution, median, and PDI measured via image analysis.


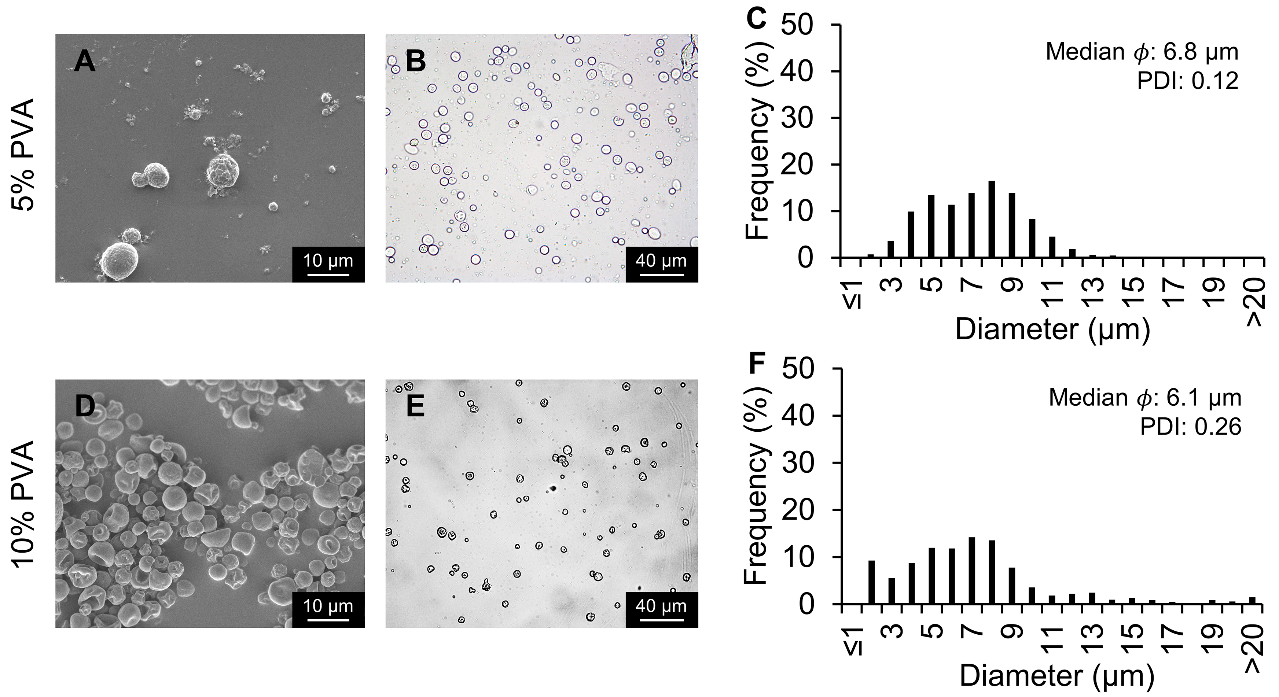


**Figure S5.** Silk particles fabricated using the 20 µm flow-focusing microfluidics device and varying the external phase PVA concentration. The internal phase flow rate was 0.8 mL/h (7% silk). The external phase concentration was either 5% or 10% PVA. (A,D) SEM and (B,E) brightfield microscopy images of silk particles. (C,F) Silk particle size distribution, median, and PDI measured via image analysis.


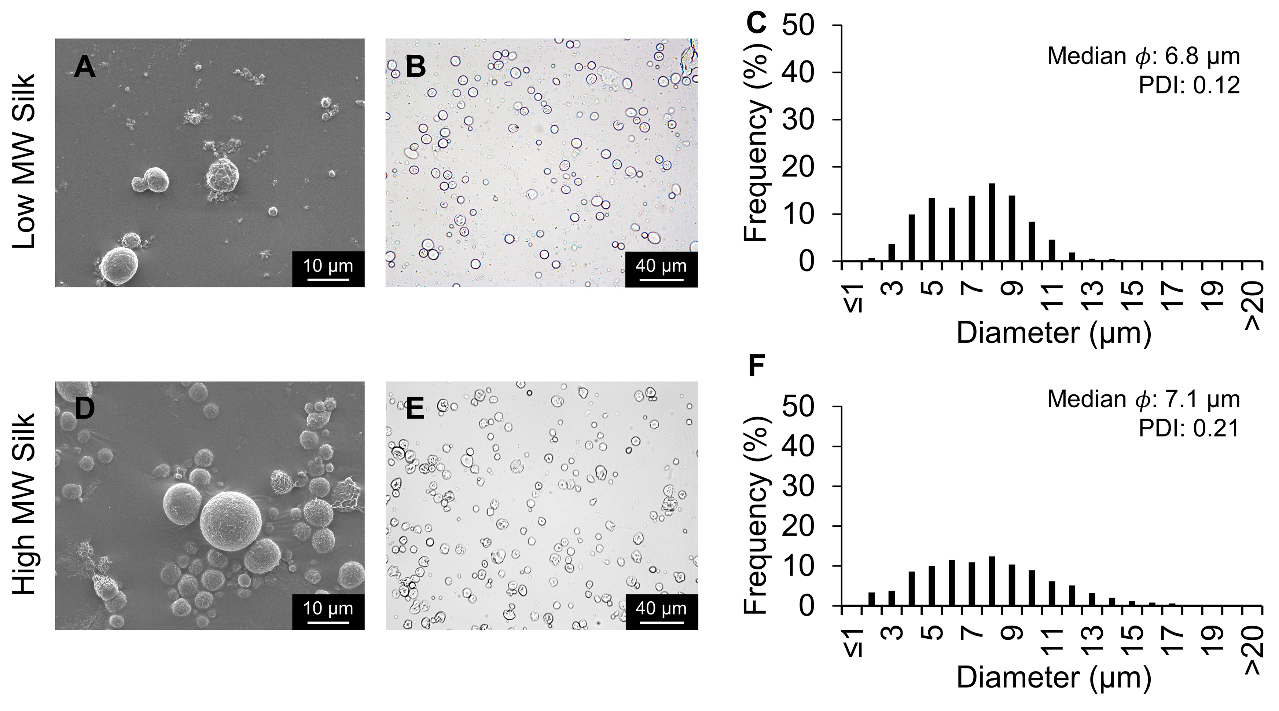


**Figure S6.** Silk particles fabricated using the 20 µm flow-focusing microfluidics device and varying the silk molecular weight via varying the extraction time. Low molecular weight silk was achieved via extracting for 120 minutes; high molecular weight silk was achieved via extract for 30 minutes. The internal phase flow rate was 0.8 mL/h. The external phase concentration was 5% PVA. (A,D) SEM and (B,E) brightfield microscopy images of silk particles. (C,F) Silk particle size distribution, median, and PDI measured via image analysis.


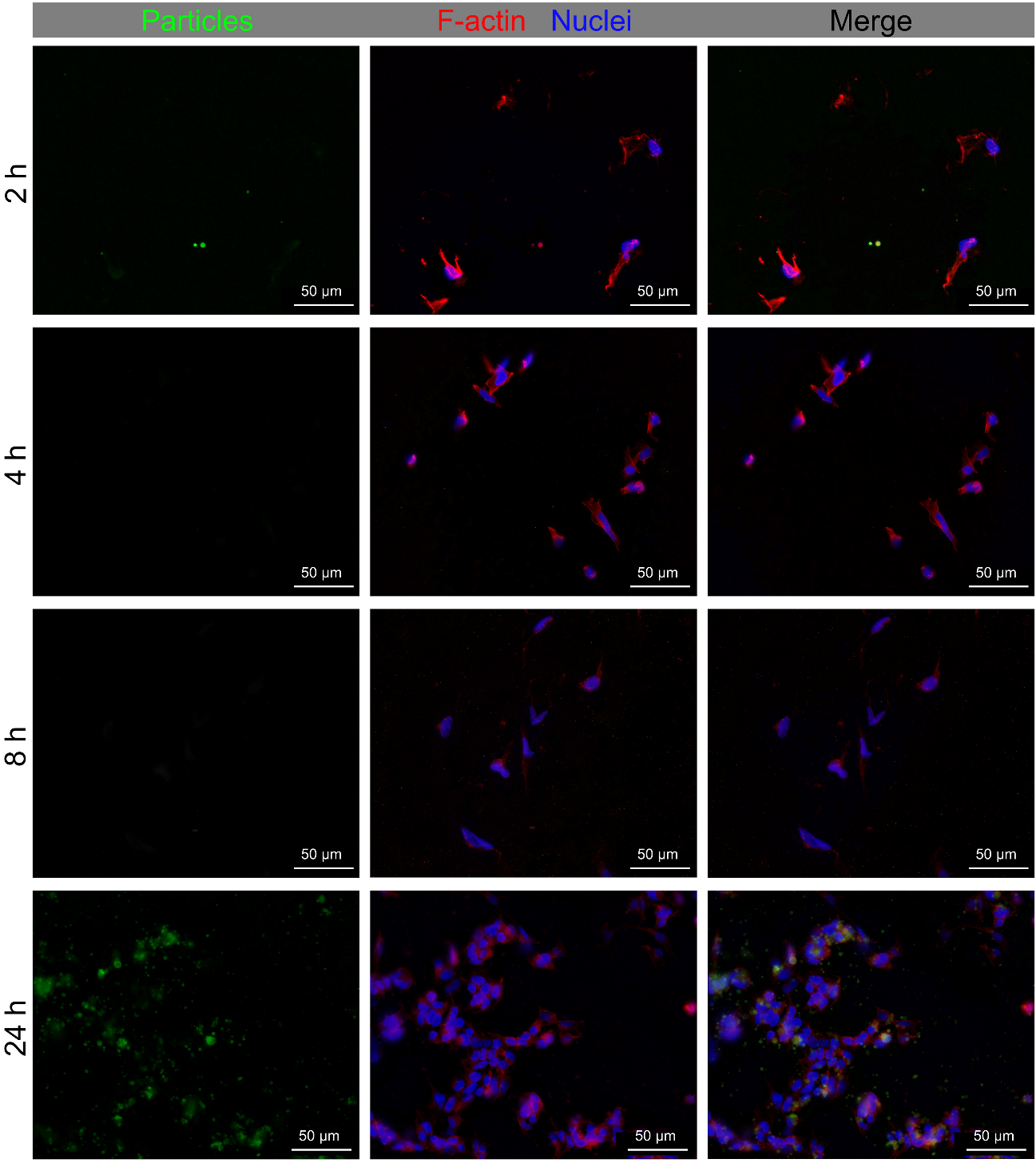


**Figure S7.** Fluorescent microscopy images of KELLY cells exposed to silk particles through 24 h.


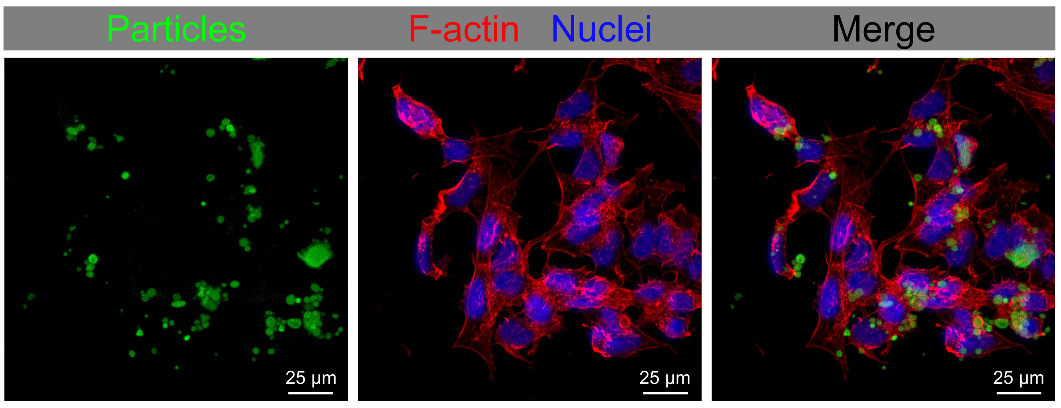


**Figure S8.** Confocal microscopy 3D projection images of KELLY cell exposed to silk particles for 24 h.


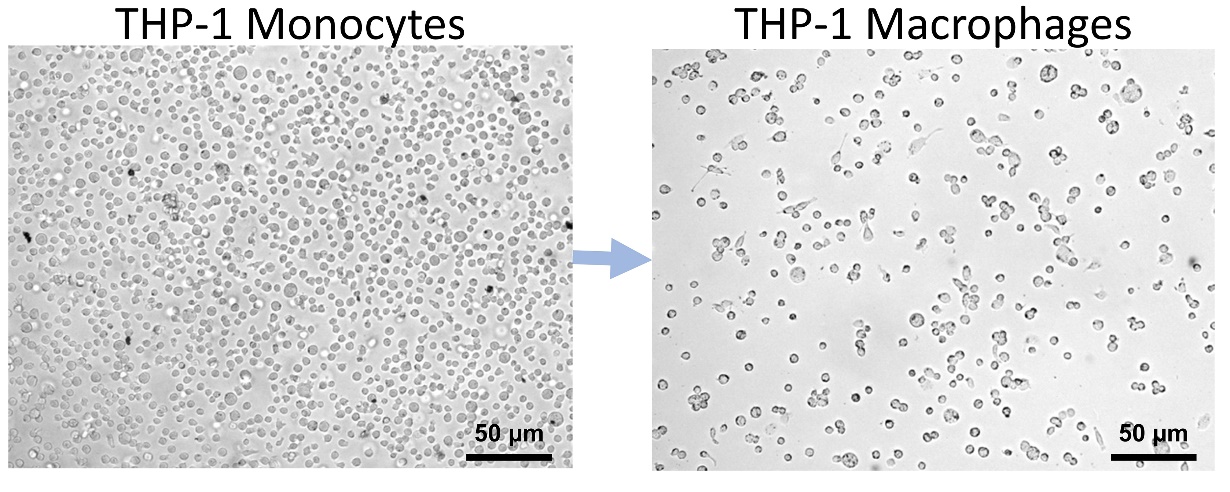


**Figure S9.** Brightfield images of the THP-1 monocyte cell line and THP-1 derived macrophages.


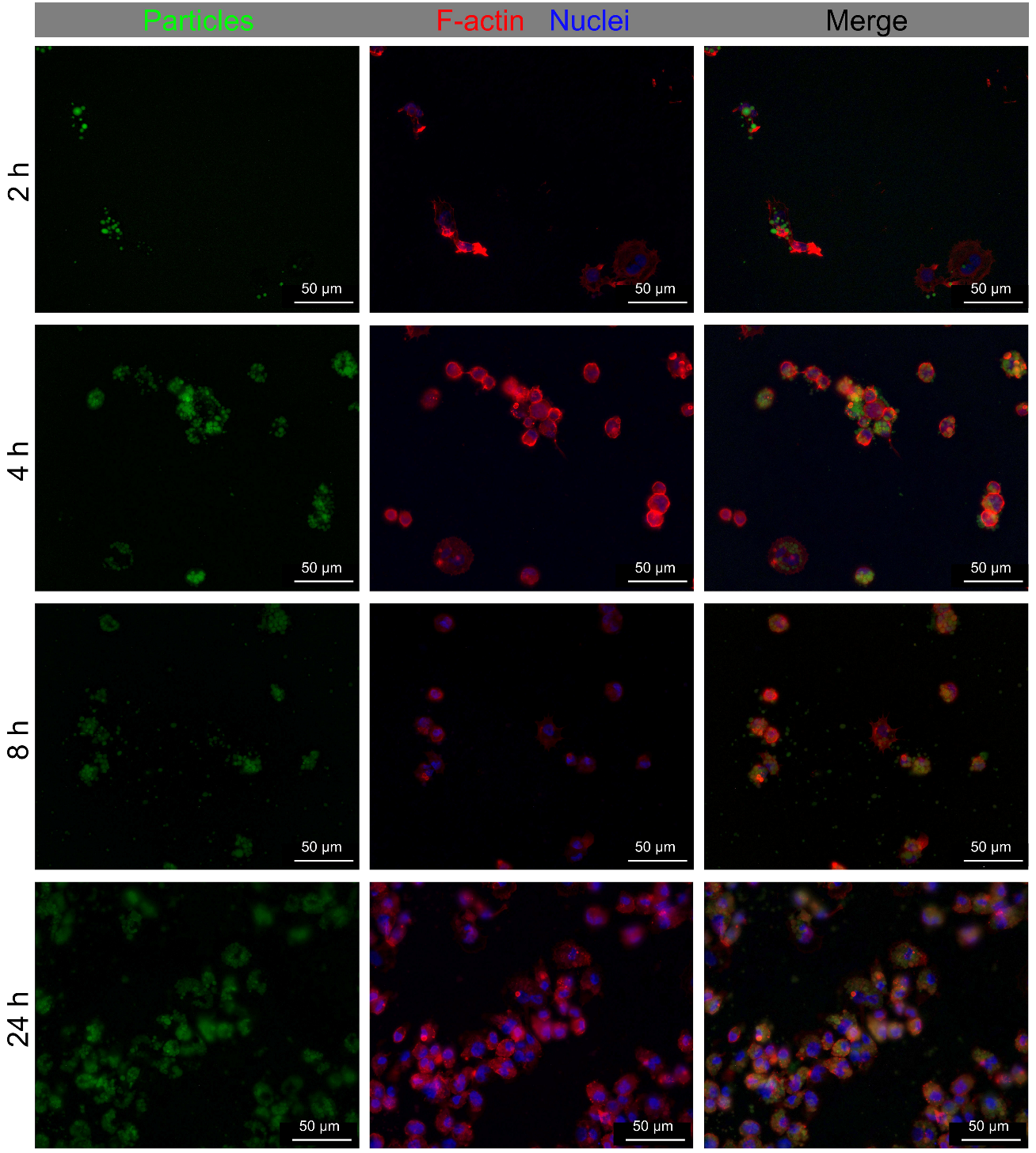


**Figure S10.** Fluorescent microscopy images of THP-1 derived macrophages exposed to silk particles through 24 h.
